# Fontanel Size from Birth to 24 Months of Age in Iranian Children

**Published:** 2015

**Authors:** Mohammad ESMAEILI, Marjan ESMAEILI, Fatemeh GHANE SHARBAF, Shirin BOKHARAIE

**Affiliations:** 1 Pediatrician, Ghaem Medical Center, Mashhad University of Medical Sciences, Mashhad, Iran; 2 Pediatrician, Iran university of Medical Sciences, Tehran, Iran; 3 Pediatrician, Dr. Sheikh Children Hospital, Mashhad University of Medical Sciences, Mashhad, Iran; 4 Mashhad University of Medical Sciences, Mashhad, Iran

**Keywords:** Fontanel, Infants, Children, Iran

## Abstract

**Objective:**

Diagnosis of abnormal fontanel size, a potential clue to recognition of different disorders, requires an understanding of the wide variation of normal fontanel size. The anterior fontanel is the largest, prominent and most important for clinical evaluation. The aim of this study was to establish and define normal range of fontanel size from birth to 24 months of age in healthy Iranian children that might be generalized to other populations.

**Materials & Methods:**

Totally, 550 subjects enrolled randomly in this cross sectional study. They were apparently normal healthy children, from birth to 24 months of age, including 208-term newborn and 342 infant from birth to 2 yr old. Fontanel size was measured and recorded as the mean of the length (anterior- posterior dimension) and width (transverse dimension). Mean anterior fontanel sizes in our samples were classified for periods of 3 months. Nomograms and statistical analyses were performed and depicted by Excel Microsoft Office 2007 and two-tailed t-test respectively.

**Results:**

The mean ±2SD of anterior fontanel size was 2.55±1.92 cm in newborns, 3.37±2.48 (largest size) in 3 months of age. It was closed in all cases in 15-18 months of age. The mean posterior fontanel size was 0.8 cm in newborns and closed in all infants in 2 months of age. There was no significant difference in anterior fontanel size between two genders except in newborn and 6-9 months old (P>0.05).

**Conclusion:**

Abnormal fontanel can indicate a serious medical condition. Therefore, it is important to understand normal variations, to utilize standardized techniques for measurement and appropriate standards of normal range in different age groups and populations. This study provides a normal range of mean fontanel size in Iranian infants as a local reference. It might be generalized to other populations.

## Introduction

The diagnosis of an abnormal fontanel e.g., bulging, sunken fontanel, large size, small size, early closure and delay closure requires an understanding of the wide variation of normal fontanel. Fontanel might be as a clue to recognition of different disorders. The sutures and fontanels in the normal skull allow that bones of the skull overlap each other at the labor time and allow brain growth continue contemporary with skull bone growth (1). Fetal and prenatal development of anterior fontanel maybe considered as an index of cranial growth (2, 3). Although anterior fontanel (AF) measurement has not been routinely as a part of the newborn examination, in infancy period it is noticed by pediatricians. The most previous studies in other countries reported normal range for fontanel dimensions only on the first days of life of the newborn infants (4-6), presented various local reference range (7-9), even in premature neonates and during various gestational age (3, 10). However, there is limited data on later infancy period (11-13) and no published report of AF size in Iranian infants between 0-24 months of age is present. The purpose of this study was to establish and define normal range of fontanel size in healthy Iranian children from the birth time to 24 months of age in northeast of Iran that might be utilized and generalized to nationally and other populations.

## Materials & Methods

**Fig 1:**
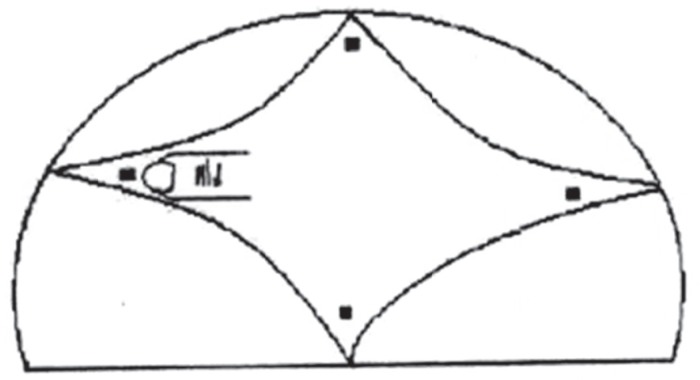
This figure shows measurement of anterior Fontanel size in the neonate

**Fig 2:**
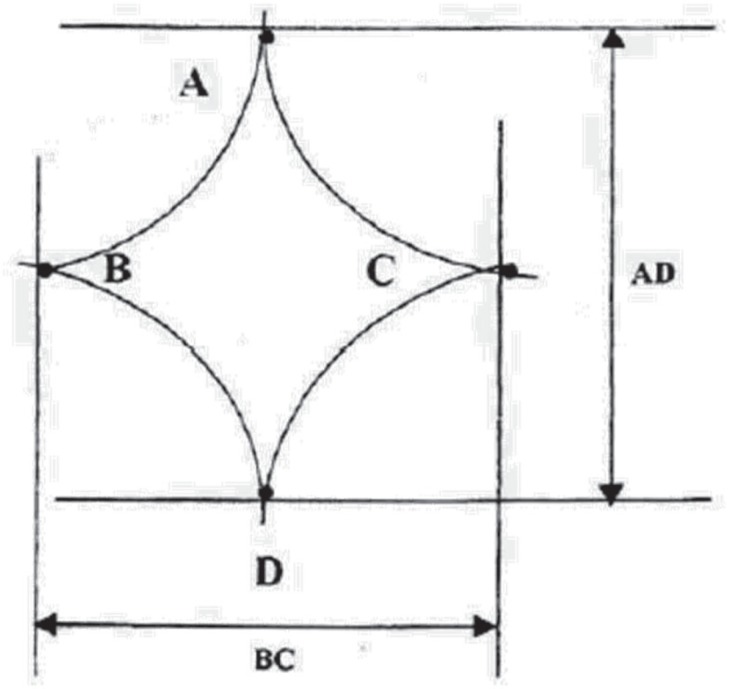
This graph shows the method use for calculation of the mean size of the anterior fontanel $\frac{\mathrm{AD}+\mathrm{BC}}{2}$ (Method of Popich and Smith)

Subjects enrolled in this cross sectional research were 550 apparent healthy children aged from 1 day to 24 months including 208 term newborn infants (110 males and 98 females) and 342 children from 1 month to 2 yr old (186 males, 156 females). Measurements were taken in the maternity ward about full term newborns within the first day of life and in hygiene and vaccination centers about children aged less than 2 yr old at the Mashhad University Medical Centers, Iran during a six months period from May to October 2006. Samplings were done by active observational method, randomly one day for every week. Gestational age of newborns was assessed by the last menstrual period and new Ballard score. A written informed consent sheet approved by Ethics Committee of Mashhad University of Medical Sciences, was filled in by the mothers to allow for investigation on their infants. Inclusion criteria were newborn with gestational age between 37-42 weeks as determined by maternal dates and Ballard score, birth weight more than 2500 gr, children less than 2 yr old age apparently normal health, without obvious mental and physical disorders or malformations. Fontanel dimensions were measured and recorded as the average of the length (anterior- posterior dimension) and width (transverse dimension) same as the method of Popich and Smith (5). To circumvent the problem of the fontanel ended and the suture began, the extent of the anterior fontanel was determined by inserting the index finger of the examiner into each of the fontanel corners and a small dot was marked with washable ink of pencil (7) (Figure 1, 2). Length of posterior fontanel was measured from the anterior corner to the midline point and the side opposite created by the occipital bone. For each infant width and length were measured with a fresh steel ruler. Mean fontanel sizes in our samples were classified for periods of 3 months. At the same time we studied effect of type of delivery and vitamin D administration on fontanel size. Statistical analysis was performed by two tailed student t-test. P-value less than 0.05 were considered statistically significant. Drawing of tables and nomogram were performed and depicted by software Excel, Microsoft office 2007.

## Results

**Fig 3:**
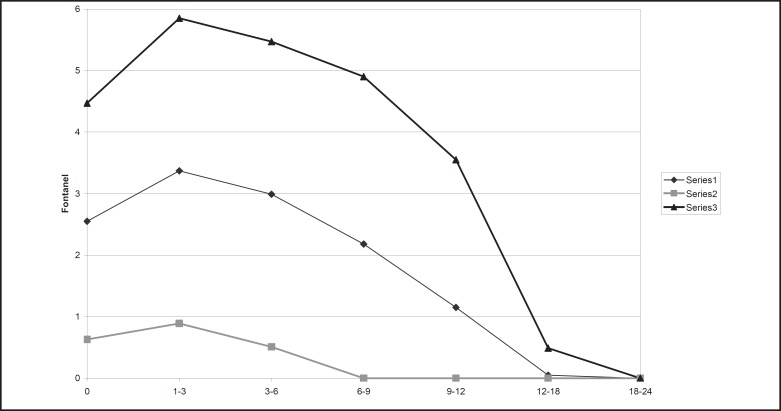
The figure depicts mean ±2 standard deviation of anterior fontanel size during first 2 years of life

**Fig 4:**
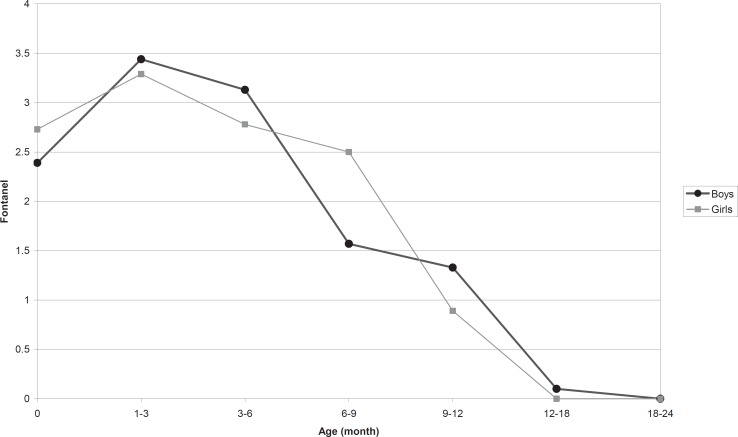
This figure depicts mean of anterior fontanel size in infant boys and girls during first 2years of life

The mean with 2 standard deviation of anterior fontanel size for newborns was 2.55±1.92 cm (range 0.55 to 4.6 cm), for 3 months of age 3.37±2.48 (range 0.8 to 6.9 cm) that is the largest fontanel size in our children. In 6% of our infants aged 6-9 months, there was closed anterior fontanel and in 94%, it was open. In 18 months of age all of the cases had closed anterior fontanel. For children less than 2 yr old age, mean ±2 SD of anterior fontanel size in all age groups old are shown in Table 1 and Figure 3. The posterior fontanel was open in 3 newborn (2% of newborns with mean size 0.8 cm) and closed in all cases in 2 months age. There was no significant difference in anterior fontanel size between boys and girls in all age groups less than 2 yr old except in newborn and 6-9 months old (Table 2 and Figure 4). Relative frequency of closed anterior fontanel is shown in Table 3. Mean ±2SD of anterior fontanel size in 98 newborn girls including 70 vaginal delivery and 28 caesarian section was 2.42±1.62 cm and 2.84±2.14 cm, respectively, so the difference was statistically significant (P=0.04). Mean ±2 SD of anterior fontanel size in 110 newborn boys including 64 vaginal delivery and 46 caesarian section was 2.32±1.7 cm and 2.47±1.82 cm, respectively without significant difference (P=0.31). The mean ±2 SD of anterior fontanel size in 98 children between one month and two yr old receiving oral vitamin D, 400IU daily and 73 without usage of vitamin D was 1.7±3.46 and 2.51±3.02 that was different significantly (P=0.002).

**Table 1:** This table shows mean of anterior fontanel size during first 2 years of life (cm)

**Age (month)**	**Number**	**Mean of Fontanel**	** Sd**
0	208	2.55	0.96
1-3	62	3.37	1.24
3-6	98	2.99	1.24
6-9	58	2.18	1.36
9-12	56	1.15	1.2
12-18	36	0.05	0.22
18-24	32	0	0
Total	550	2.24	1.47

**Table 2:** This table demonstrates comparison between mean of anterior fontanel size during first 2 years of life in Boys and girls (cm)

**Age (month)**	**Number**		**Mean of Fontanel**		**Sd**		***P*** **-Value**
	**Boy**	**Girls**	**Boy**	**Girls**	**Boy**	**Girls**	
0	110	98	2.39	2.73	0.86	1.02	0.01
1-3	34	28	3.44	3.29	1.18	1.39	0.63
3-6	60	38	3.13	2.78	1.19	1.3	0.11
6-9	24	34	1.57	2.5	1.15	1.43	0.01
9-12	28	28	1.33	0.89	1.38	1.04	0.10
12-18	20	16	0.1	0	0.31	0	0.19
18-24	20	12	0	0	0	0	1

**Table 3:** This table shows frequency of closed anterior fontanel during infancy period

**Age (month)**	**Number**	**Closured Fontanel**	**%**
<7	386	0	0
7-8	30	2	6
8-9	12	2	16
9-10	20	4	20
10-11	18	4	22
11-12	18	14	77
12-15	12	10	83
15-18	24	24	100
>18	32	32	100

## Discussion

Examination of an infant’s fontanels offers the physician a window into the infant developing brain and general state of health. The normal fontanel varies widely in shape and time of closure. A newborn has actually six fontanels, the anterior one, which is the largest and most important clinically, posterior one, two mastoid and two sphenoid. Apparently, an infant has two prominent fontanels at birth: a diamond-shaped anterior fontanel at the junction of the frontal and parietal bones that is open at birth, and a triangular posterior fontanel at the junction of the parietal and occipital bones that can admit the tip of a finger or may be closed at birth. If the posterior fontanel is open at birth, it should close over the ensuing 6-8 wk; its persistence suggests underlying hydrocephalus or congenital hypothyroidism. The anterior fontanel varies greatly in size. The average time of closure is 18 mo, but the fontanel can close normally as early as 9 mo. A very small or absent anterior fontanel at birth might indicate craniosynostosis or microcephaly, whereas a very large fontanel can signify a variety of problems. The fontanel is normally slightly depressed and pulsatile and is best evaluated by holding the infant upright while he or she is asleep or feeding. A bulging fontanel is a reliable indicator of increased ICP, but vigorous crying can cause a protuberant fontanel in a normal infant (14- 15). The average size of the anterior fontanel is 2.1 cm and the median time of closure is 13.8 mo in oldest reports (1) which were approximate to our results. Popich and Smith enumerated a variety disorders in which abnormal fontanel size may be one feature (5). In our study, the anterior fontanel size of the term newborns (2.55±1.92 cm) was different from those of Popich and Smith (2.1±1.5 cm) and Faix study (4) (2.1±1.5 cm). The most likely explanation may be due to different racial and different time of measuring in first days of life. The geometry of the index fingertip may introduce some variability to the methods used in these studies. Growth of cranium is triggered by brain growth, two third of which occurs by two years of age. The sutures remain open until brain growth ceases in the second decade of life. Therefore, fontanel size is influenced by brain growth, dural attachments, suture development and osteogenesis (15). The newborn’s skull is molded during delivery through the birth canal and usually resolved after three to five days. A limitation of our study may be examination of fontanel in first 24 h of life because of routine hospital discharge of healthy newborns that cannot be reliable predictor of fontanel size at next days. Embriologically the anterior fontanel is defined laterally by the bony skull plate and cannot be delineated until 12 weeks’ gestation when these first appear. The most important feature is the development of an elastic membrane in the connective tissue below the skin by 23 weeks’ gestation and a subcutaneous layer of fat that appears by 28 weeks’ gestation. The eventual closure of the anterior fontanel is thought to occur by transformation of fibroblasts into osteoblasts after birth. These form bony islands that join to the main skull plate (16). The actual size of the fontanel increases during gestation, while its size in relation to the volume of the fetal head diminishes, possibly due to the rapid development of the brain hemispheres and the consequent outward growth of the calvarial bones. The fact that enlarged fontanel dimensions may be associated with certain fetal abnormalities may be employed advantageously in the differential diagnosis of some syndromic conditions in utero (2). There is scanty data in the literature on the variation of fontanels size with gestational age. This relationship was studied at the University College Hospital, Ibadan, Nigeria. Anterior fontanel size showed a low positive correlation with gestational age (r = 0.15). The posterior fontanel size did not show a significant correlation with gestational age and the prevalence of closed posterior fontanels at birth was not different significantly between term and preterm neonates (10). The posterior fontanel is normally less than 1 cm at the time of birth and is no longer palpable by 8 weeks. A posterior fontanel that feels larger than expected should alert the provider to all the conditions described herein that could also cause an enlarged anterior fontanel. An additional genetic cause of an increased posterior fontanel includes parietal foramina syndrome (17). Some investigators (18) have measured size and area of posterior fontanelle, as well as its relations with gestational age in human fetuses. Their study was aimed to provide a range of normal posterior fontanelle dimensions and to provide base for further research in Asian population on morphology of posterior fontanelle and see if there exists any significant difference compared to European & African studies. Fifty fetuses were arranged in 5 groups according to their gestational age. Mean posterior fontanelle size and area of each group was measured. It was observed that posterior fontanelle size and area both vary significantly with gestational age (18). The fontanels should be examined while the infant is calm and held in both supine and upright position. In early life, the anterior fontanel is open and may be useful to evaluate ventricles by ultrasound and neuroimaging procedures. In infants, the fontanels and sutures as well as conductivity of the skull influence the volume currents accompanying primary currents generated by active neurons and thus the associated electroencephalography (EEG) and magnetoencephalography (MEG) signals (19). There are various methods of measurement described as simple clinical methods of measuring anterior fontanel (7, 20-21). Jackson et al. sought to determine anterior fontanel size (AFS) in Hispanic neonates and to compare two methods of measurement (20). The traditional method (TRAD) was defined as the sum of the longitudinal and transverse dimensions, divided by 2. Diagonal measurements (DIAG) were obtained between the estimated midpoints of the edges of the frontal and parietal bones, and the sum was divided by 2. Measurements by TRAD and DIAG (mean ± standard deviation) were 22.5 ± 7.9 mm and 20.9 ± 6.7 mm, respectively (P = 0.12). AFS was greater in males and in neonates, whose mothers had longer duration of labor. A modest trend toward less variability with the DIAG method was noted. Male gender and longer duration of labor were associated with larger AFS (20). In our study, we used TRAD method, as a simple method. Normal anterior fontanel has a wide variation. In Zurich, size and closure of the anterior fontanel from birth to 24 months of age and their relationships to growth parameters, bone age, and gestational age were reported. Great variability of both fontanel size and age when fontanel closed was observed. No significant relationships were noted between anterior fontanel size and head circumference or bone age (20). In the last decades, there was very limited information for detection of fontanel size in the first two years of life (12, 23), whereas our research has evaluated this enigma. Because there is no documented study in our population in this age group, our data might help to establish a pattern of normality. In Iranian neonates, a significant difference between the mean anterior fontanelle size in boys and girls was found (P=0.023). There was no significant difference in anterior fontanelle size between the infants born with a normal vaginal delivery and those with cesarean section (P=0.08). There was found a significant negative correlation between the mean size of anterior fontanelle size with both weight and height (P<0.05). No significant correlation was found between mean size of anterior fontanelle and head circumference or with gestational age of infant (P≥0.05) (7). Our study showed mean anterior fontanel size in neonate and 6-9 mo old girls, which was larger than that of boys. Black neonates have larger fontanel (1.4 to 4.7 cm) in comparison with Caucasians newborns. However the racial difference should be appreciated when assessing fontanel size that are greater for blacks than for whites (4, 24). The mean AF size in neonates was larger than published figures from Caucasian and Chinese populations, but at 12 months mean AF size in Nigerian infants was smaller than published Caucasian and Chinese figures (24). Our study showed anterior fontanel size in newborn as 2.55±1.92 cm (range 0.55-4.6 cm). It had the largest size in 3 mo aged (3.37±2.48 yr) and closed in all cases in 15-18 months of age, same as previous studies in Iranian newborns, Caucasian and Arab infants ( 5- 7,9), but was smaller than black infants (24) and larger than Scottish neonates (11). In our investigation, posterior fontanel was closed in all cases in 2 mo aged, with mean size of 0.8 cm at birth time. In another study, at birth the average size of posterior was 0.5-0.7 cm, usually is completely closed by 2 months of age (4), which was in agreement with our study. Until this time, there were few studies on effect of type of delivery on fontanel size. The study of Pedroso et al. in Brazil showed disagreement with literature data, fontanel size increased up to 2 months of age; fontanel was closed at 1 year for 27.3% of infants. Significant differences in cranial anthropometry were not found in relation to method of delivery (vaginal or cesarean section), gender and gestational age (21). In contrast to previous studies (7, 21), in our study the type of delivery had effect on anterior fontanel size especially in female neonates, that was significant statistically. Since vitamin D usage prevents rickets and osteopenia, in our study the anterior fontanel size in those that administered vitamin D was smaller than those without usage of vitamin D. Delayed closure of the anterior fontanelle can be associated with multiple diseases, most of which have dysmorphic features that should facilitate early recognition. Increased intracranial pressure is the most common cause of delayed closure of the anterior fontanelle (17, 25). Many skeletal disorders are responsible for delayed closure of the anterior fontanelle. Achondroplasia, osteogenesis imperfecta, vitamin D deficiency–rickets, and cleidocranial dysostosis are the most common cases. These diseases present with characteristic physical findings and are confirmed by associated laboratory and x-ray abnormalities. Chromosomal defects as well as dysmorphogenetic syndromes also predispose infants to delayed closure of the anterior fontanelle. Down, trisomy 13, trisomy 18, Russell- Silver, RubinsteinTaybi, and Robinow’s syndromes commonly encompass this physical finding within the constellation of findings associated with that particular syndrome. Endocrine disorders as well as drug and toxin exposure are also associated with delayed closure of the anterior fontanelle. Commonly, hypothyroidism, fetal hydantoin syndrome, aminopterin-induced malformations, and aluminum toxicity can all be associated with a persistent open fontanelle. These also are easily ruled out via a thorough history, blood levels, and thyroid function screening (15- 17, 26). A bulging anterior fontanel can be a result of increased intracranial pressure, hydrocephalus and tumor. Clinical evaluation of fontanel gives good guidance to assess the status of hydrocephalus in early childhood (27). A sunken fontanel is sign of dehydration. However fontanel closure that occurs as early as three months of age can be within normal limit, but careful monitoring of head circumference in such cases is essential to exclude a pathologic condition. Craniosynostosis and abnormal brain development are associated with a small fontanel or early fontanel closure (28-29).

**In conclusion**, abnormal fontanel in an infant can indicate a serious medical condition; therefore, it is important to understand the wide variation of the normal fontanel size and shape in different racial groups. To assess anterior fontanel size properly, standardized techniques for measurement and appropriate use of standards nomograms should be carried out. This study provides the normal range of mean fontanel size in Iranian infants as a local reference and might be generalized to other population. However, a longitudinal cohort investigation using local and multicenter studies maybe more reliable.
